# Genome mining of *Mycobacterium tuberculosis*: targeting SufD as a novel drug candidate through *in silico* characterization and inhibitor screening

**DOI:** 10.3389/fmicb.2024.1369645

**Published:** 2024-04-15

**Authors:** Neelima Gorityala, Anthony Samit Baidya, Someswar R. Sagurthi

**Affiliations:** Department of Genetics and Biotechnology, Osmania University, Hyderabad, Telangana, India

**Keywords:** *Mycobacterium tuberculosis*, subtractive genomics, iron-sulfur cluster assembly, SufD, inhibitor screening, SufD expression and purification

## Abstract

Tuberculosis (TB) stands as the second most fatal infectious disease globally, causing 1.3 million deaths in 2022. The resurgence of TB and the alarming rise of antibiotic resistance demand urgent call to develop novel antituberculosis drugs. Despite concerted efforts to control TB, the disease persists and spreads rapidly on a global scale. Targeting stress response pathways in *Mycobacterium tuberculosis (Mtb)* has become imperative to achieve complete eradication. This study employs subtractive genomics to identify and prioritize potential drug targets among the hypothetical proteins of *Mtb*, focusing on indispensable pathways. Amongst 177 essential hypothetical proteins, 152 were nonhomologous to human. These proteins participated in 34 pathways, and a 20-fold enrichment of SUF pathway genes led to its selection as a target pathway. Fe–S clusters are fundamental, widely distributed protein cofactors involved in vital cellular processes. The survival of *Mtb* in a hypoxic environment relies on the iron–sulfur (Fe–S) cluster biogenesis pathway for the repair of damaged Fe–S clusters. It also protects pathogen against drugs, ensuring controlled iron utilization and contributing to drug resistance. In *Mtb*, six proteins of Fe–S cluster assembly pathway are encoded by the *suf* operon. The present study was focused on SufD because of its role in iron acquisition and prevention of Fenton reaction. The research further delves into the *in silico* characterization of SufD, utilizing bioinformatics tools for sequence and structure based analysis. The protein’s structural features, including the identification of conserved regions, motifs, and 3D structure prediction enhanced functional annotation. Target based virtual screening of compounds from the ChEMBL database resulted in 12 inhibitors with best binding affinities. Drug likeness and ADMET profiling of potential inhibitors identified promising compounds with favorable drug-like properties. The study also involved cloning in SUMO-pRSF-Duet1 expression vector, overexpression, and purification of recombinant SufD from *E. coli* BL21 (DE3) cells. Optimization of expression conditions resulted in soluble production, and subsequent purification highlighting the efficacy of the SUMO fusion system for challenging *Mtb* proteins in *E. coli*. These findings provide valuable insights into pharmacological targets for future experimental studies, holding promise for the development of targeted therapy against *Mtb*.

## Graphical abstract

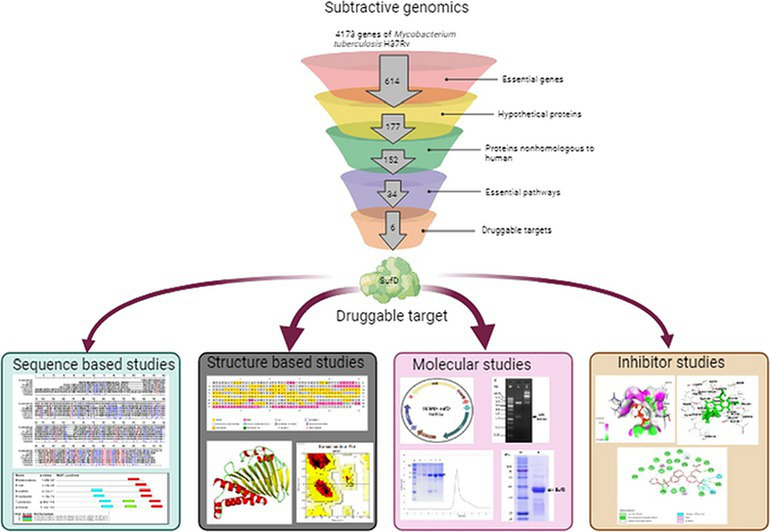

## 1 Introduction

Tuberculosis (TB) is the second leading infectious killer next to COVID-19 worldwide and claimed 1.3 million deaths in 2022 according to Global tuberculosis report, 2023 by World Health Organization (Tuberculosis, n.d.). In 2022, the infection rate spiked to 7.5 million which is the highest since WHO began TB monitoring in 1995. Despite the attempts to control TB it still continues to spread rapidly and highlights the need to elevate efforts in the fight against TB. The adaptable nature of *Mycobacterium tuberculosis (Mtb)*, manifesting through physiological changes, acquired traits, and inherent resistance pathways, contributes significantly to TB resurgence, drug resistance, and poses a substantial public health threat. Additionally, mutations acquired in genes targeted by current medications fuel the emergence of drug resistance, resulting in multidrug-resistant and extensively drug-resistant TB. Further complicating matters is the scarcity of new pharmacological targets, hampering the development of effective antitubercular compounds. Therefore development of novel and effective drugs with new biological mechanisms of action against persistent infection is the need of the hour. Approximately 25% of *Mtb* genes are annotated as hypothetical proteins (HPs), lacking confirmed functions (Yang H. et al., 2019; Yang Z. et al., 2019). Notably, certain “hypothetical” proteins in *Mtb* are predicted to play key roles in the pathogen’s intracellular lifestyle and survival in diverse environments (Yang z. et al., 2019). *In silico* methods, being more adept at handling large datasets and high-throughput analyses compared to traditional lab experiments, offer a smoother route to process and analyze vast amounts of biological data (Jo et al., 2020). Hence, our study aimed to conduct genome wide screening of essential hypothetical proteins using these *in silico* methods to identify potential drug targets. We employed subtractive genomics approach that involved examining both the pathogen and host genomes. Our focus was on isolating the distinct and vital genetic elements of the pathogen that could serve as targets for drug development. This method specifically narrows down genes absent in the host but crucial for the pathogen’s growth and persistence—referred to as “non-host” genes. The ultimate goal is to craft therapeutic compounds that selectively impact the pathogen’s metabolic activity while sparing the host’s biology. This could potentially disrupt crucial gene functions in the pathogen and diminish its ability to cause disease. This methodology has been successfully applied in various studies targeting potential drug targets in pathogens such as *Streptococcus pneumonia* (Wadood et al., 2018) *Pseudomonas aeruginosa* (Choi et al., 2002)*, Acinetobacter baumannii* (Goyal et al., 2018)*, Staphylococcus aureus* (Uddin and Saeed, 2014), and others.

In the current investigation, an essential pathway identified through a subtractive genomics approach was the SUF system, specifically the Fe–S cluster assembly pathway. Fe–S clusters are the most primitive, ubiquitous, versatile protein cofactors of a multitude of enzymes involved in crucial cellular processes (Fontecave, 2006). The disruption of Fe–S clusters not only hinders critical processes but also results in the release of iron ions. Ferrous ions react with oxygen to generate superoxide. Subsequently, superoxide undergoes dismutation, resulting in the formation of hydrogen peroxide that reacts further with ferrous ions to produce hydroxyl radicals through the Fenton reaction (Vilchèze et al., 2013). In bacteria, a prevalent mechanism of cell death induced by bactericidal drugs, irrespective of their specific target, revolves around the production of hydroxyl radicals through the Fenton reaction (Kohanski et al., 2007). Upon phagocytosis of *Mtb* by alveolar macrophages, it encounters various stresses such as acidic pH, nutrient starvation, oxidative stress, and nitrosative stress (Schnappinger et al., 2003). The immune system’s containment of infected macrophages within granulomas leads to a hypoxic environment (Aly et al., 2006; Via et al., 2008). Oxidative stress induced by the host environment poses a significant challenge to *Mtb* during infection, and this stress is aggravated by the presence of anti-TB drugs (Kim et al., 2012; Bhaskar et al., 2014). However, it maintains redox balance to survive under stress and defend themselves against drugs (Pacl et al., 2018). In response to this stress iron–sulfur cluster assembly pathway is activated which is not only responsible for assembling and maintaining Fe–S clusters but also play a protective role by ensuring the controlled and proper utilization of iron ions (Elchennawi and Choudens, 2022). Intracellular resistance of *Mtb* to oxidative and nitrosative stress is due to the upregulation of genes involved in Fe–S cluster assembly (Voskuil et al., 2011). Although reactive oxygen intermediates and reactive nitrogen intermediates cause damage to microbial DNA, lipids, and proteins, Fe–S clusters are particularly vulnerable cellular cofactors (Imlay, 2006; Jang and Imlay, 2007).

In bacteria, the three highly conserved systems identified to date for Fe–S cluster assembly are the iron sulfur cluster (ISC), sulfur formation (SUF), and nitrogen fixation (NIF) system (Ayala-Castro et al., 2008; Garcia et al., 2022). Unlike other bacteria, *Mtb* possesses only a SUF system for Fe–S cluster assembly organized in a single gene cluster named *suf* operon. This operon encodes seven contiguous genes *Rv1460 (sufR), Rv1461 (sufB), Rv1462 (sufD), Rv1463 (sufC), Rv1464 (sufS), Rv1465 (sufU), and Rv1466 (sufT)* (Huet et al., 2005; Pandey et al., 2018). Fe–S cluster assembly occurs in two main stages: First, assembly of the Fe–S cluster on the scaffold protein which receives sulfur from the cysteine using cysteine desulfurase, and the iron from an unidentified donor protein. Second, transfer of the assembled Fe–S cluster to the apoprotein using ATP-hydrolyzing component. The distribution and organization of these systems may differ across species, but their fundamental function remains unchanged, which is to assemble and transport Fe–S clusters to apoproteins (Py and Barras, 2010).

All the genes of the *suf* operon except *sufR (Rv1460)* are anticipated to be essential for the growth of *Mtb* (Sassetti et al., 2003; DeJesus et al., 2017). Previous studies indicated that *sufR* negatively regulates the downstream genes *(Rv1461-Rv1466)* (Willemse et al., 2018). A recent study revealed that SufS is a type II cysteine desulfurase enzyme whose activity is enhanced by interaction with SufU (Elchennawi et al., 2023). Another study reported SufT as indispensable for Fe–S cluster maturation and cellular homeostasis (Tripathi et al., 2022). In *E. coli,* SufB, SufC, and SufD form the SufBC_2_D complex, which serves as a scaffold for the assembly of nascent Fe–S cluster (Hirabayashi et al., 2015). SufBC forms an interaction with SufCD within the SufBC_2_D complex, which plays a prominent role in iron acquisition during *in-vivo* Fe–S cluster assembly. A systematic mutational analysis of SufD unveiled that a functional region exists at the interface between SufB and SufD, which constitutes the site for *de-novo* cluster formation (Yuda et al., 2017). Despite extensive research, structural and functional characterization of SufB, SufC, and SufD proteins in *Mtb* remains unexplored. In the UniProt database, these proteins are predicted to be functionally necessary for Fe–S cluster assembly (*Rv1461, Rv1462*) and ATP binding ABC transporter (*Rv1463*).

Previous research has demonstrated SufD’s pivotal role in iron acquisition in *E. coli* (Saini et al., 2010). Building on these findings, we propose the hypothesis that in *Mtb,* SufD might be responsible for capturing ferrous ions released during Fe–S cluster damage, thus averting the Fenton reaction. The importance of the SufD and its central role in addressing the stress conditions faced by *Mtb* underscore its potential as an attractive target for the design of new anti-TB drugs. Thus SufD was selected as a suitable target for our study.

To assess the potential of SufD as a drug target, investigations involved both sequence and structure based studies. Structure based drug design was proven to be more efficient in drug discovery. The use of computational methods allow delivery of new drug candidates quickly and cost-efficiently thus expediting this step in drug discovery pipeline. Prediction of 3D structure for SufD enabled the detection of binding pockets and facilitated screening for inhibitors. Simultaneously, the gene was cloned, and rSufD was successfully expressed and purified in *E. coli*, overcoming challenges such as inclusion body formation. This holistic method for pinpointing pharmacological targets in *Mtb* offers valuable insights for forthcoming experimental investigations.

## 2 Materials and methods

### 2.1 Subtractive genomics

#### 2.1.1 Screening and identification of essential, hypothetical, non-homologous proteins

We employed the Database of Essential Genes (DEG) to identify the genes that are essential and annotated as Hypothetical Proteins (HPs) within *Mtb* genome (Zhang et al., 2004; Luo et al., 2021). Understanding these bacterial HPs aids in pinpointing potential new targets for drug development. Proteins vital to pathogens, bearing no similarity to host proteins, represent promising drug targets. Hence, the essential hypothetical protein sequences of *Mtb* underwent BLASTp against the *Homo sapiens* proteome, setting an expectation value (*e*-value) of 10^−3^. In subsequent analysis, only *Mtb* proteins with no human homologues were considered for the study.

#### 2.1.2 Essential pathway analysis

**Figure 1:**
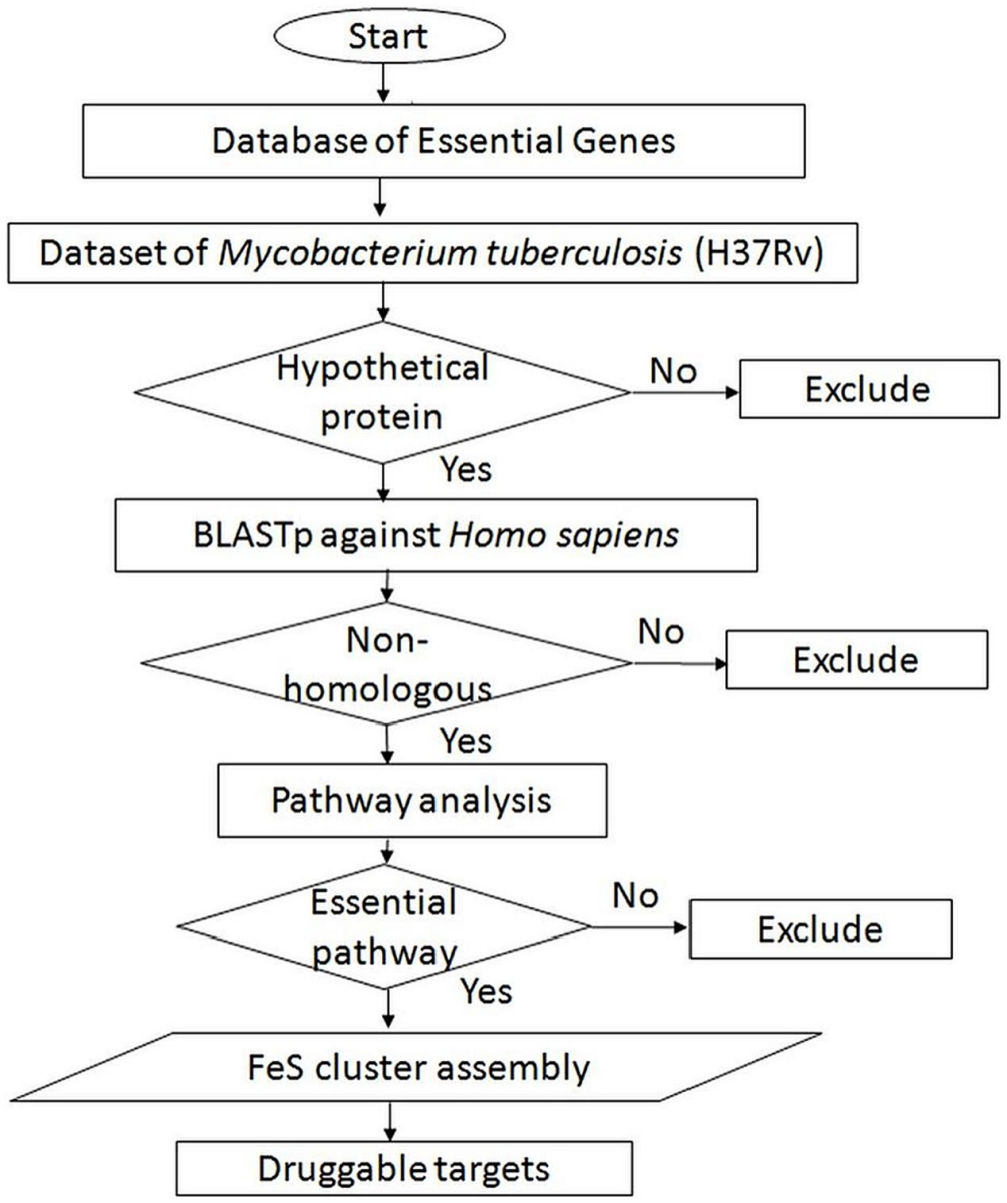
Schematic of subtractive genomic analysis for screening of potential novel druggable targets from *Mycobacterium tuberculosis*.

The distinctive hypothetical proteins exclusive to *Mtb* were subjected to pathway prediction using the ShinyGO v0.75 server (Ge et al., 2020). ShinyGO is a user-friendly graphical web application designed to extract actionable insights from sets of genes. Through enrichment analysis, this tool connects gene lists with the associated molecular pathways and functional categories, including gene ontology (GO) and other relevant databases. An overview of the subtractive genomics approach is illustrated in Figure 1.

### 2.2 *In silico* characterization of druggable target

#### 2.2.1 Comparative protein analysis and motif identification in SufD

We used the online BLAST tool to assess the conservation of proteins homologous to SufD. BLASTp was executed with UniProtKB as the target database, using default settings. The homologous sequences were then subjected to multiple sequence alignment (MSA) using the Multalin tool to compare the conservation of the SufD protein across various species (Corpet, 1988). Additionally, we used the MEME suite to analyze the sequences and identify conserved motifs (Bailey et al., 2015). The MEME Suite web server provides a comprehensive platform for online discovery and analysis of sequence motifs, including features such as DNA binding sites and protein interaction domains.

#### 2.2.2 Prediction of subcellular location

The subcellular location of SufD protein was determined using two tools: PSORTb v3.0 and CELLO2GO (Yu et al., 2010, 2014). These tools help identify different compartments within a cell where proteins can reside, such as the cytoplasmic membrane, cytoplasm, cell wall, extracellular and unknown regions.

### 2.3 Structure based studies

#### 2.3.1 Homology model building and structure evaluation

The SufD protein sequence was used as an input for modelling through the I-TASSER server. The modelling process begins with identifying structural templates from the Protein Data Bank (PDB) using the LOMETS threading approach (Zhang et al., 2004; Roy et al., 2010; Yang et al., 2015). Only the highest significance templates from the threading alignments are used. The I-TASSER simulations produce various structural conformations called decoys. These decoys are then clustered using the SPICKER program to determine the final models. The C-score is used to assess the confidence of each model quantitatively. The structure with the highest C-score is considered for further evaluation.

To evaluate the SufD model’s structure, secondary structure analysis was done using PSIPRED, while PROCHECK was used for tertiary structure stereochemistry analysis (Laskowski et al., 1993; Buchan and Jones, 2019).

#### 2.3.2 Structure based function prediction

The generated model of the SufD structure was uploaded to the ProFunc server for a comprehensive evaluation of the structure and the predicted function of the protein (Laskowski et al., 2005). The primary goal of the ProFunc server is to assist in discerning the probable biochemical function of a protein based on its three-dimensional structure. The server employs various methods, such as sequence searches, residue conservation analysis, surface cleft examination, and the utilization of functional 3D templates. These approaches collectively aim to identify the likely active site of the protein and potential homologues present in the PDB.

#### 2.3.3 Prediction of binding pockets

The Computed Atlas of Surface Topography of Proteins (CASTp) server was used to identify the binding pockets of the protein. The protein structure file was uploaded in pdb format (Tian et al., 2018). The CASTp server analyzed the protein structure and predicted potential binding pockets based on characteristics such as solvent accessibility, geometric shapes, and surface areas. The credibility of the cavities that were forecasted was further assessed using the SCFBio and Depth active site prediction web servers (Jayaram et al., 2006, 2014; Tan et al., 2013).

### 2.4 Target based screening and inhibitor identification

#### 2.4.1 Compound dataset and ligand preparation

A search was conducted in the Chemical Database at European Molecular Biology Laboratory (ChEMBL) database for bioactive molecules that are likely to bind with Rv1462 (SufD). A total of 147 molecules were found by searching the term “Rv1462.” Among 147 molecules, the compounds with highest binding affinity to SufD were considered for further studies while rest of them may bind with other proteins and enzymes. The LigPrep module of the Schrödinger suite, 2013 was used to optimize all the ligands through the OPLS 2005 force field algorithm (Schrödinger Release 2023-4: LigPrep, 2024). The molecules were then prepared for docking using Marvin Sketch and its allied applications, which were used to add explicit hydrogens, clean the molecule in 3D, and prepare it for docking (Marvin 5, 2011). The ligand ionizations were desalted and retained at the original state. To serve as a control, molecule 882 (CHEMBL1539876), which is a proven inhibitor of Fe–S cluster biogenesis in *Staphylococcus aureus (S. aureus)*, was used (Choby, 2016).

#### 2.4.2 Virtual screening

Molecular docking program- Molegro Virtual Docker (MVD 2012.5.5) integrated with MolDock scoring function was used to dock the compounds in the predicted active site of Rv1462 (Thomsen, 2006). Docking parameters were set with the Moldock SE search algorithm with a maximum docking iteration of 1,500, at a grid resolution of 0.20 Å, with a maximum population size of 50. A maximum of 5 poses were generated for each ligand using simplex evolutions with a default parameter of 300 maximum steps with a neighborhood distance factor of 1.0. Binding affinity and interactions of inhibitor with the protein were evaluated based on the sp2-sp2 torsions, internal hydrogen bond interactions, and internal electrostatic interactions.

#### 2.4.3 Protein ligand interactions

To study the interactions between SufD and a potential ligand, we conducted an analysis using BIOVIA Discovery Studio 2020 Client. We imported the chosen ligand and the protein pose obtained during the docking process into Discovery Studio. This helped us visualize the molecular interactions, including hydrogen bonding, hydrophobic interactions, and other relevant interactions (BIOVIA, 2020).

#### 2.4.4 ADMET profiling and drug likeness of selected ligands

We used the admetSAR tool to analyze the chemical descriptors and druggability of the top-docked compounds (Yang H. et al., 2019). This tool helped predict various pharmacokinetic parameters, including absorption, distribution, metabolism, excretion, and toxicity. We utilized endpoints such as Ames, carcinogenicity, hepatotoxicity, and skin sensation to assess the toxicity of the selected compounds. Furthermore, the prediction of drug-likeness properties involved the application of specific rules based on compound structural properties. For the evaluation of the drug-likeness of compounds, the open-source virtual screening tool DruLiTo was utilized (Brenk et al., 2008). Various parameters were computed, covering Molecular Weight (MW), logP, AlogP, H-Bond Acceptor and Donor (HBA and HBD), Total Polar Surface Area (TPSA), Atom Molar Refractivity (AMR), number of Rotatable Bonds (nRB), number of Acidic groups, number of Rigid Bonds (nRigidB), Rotatable Bond Count (RC), nAtom Ring, and nHB. The tool evaluated drug-like characteristics according to criteria such as Veber’s rule, Lipinski’s rule, CmC-50-like rule, MDDR-like rule, BBB rule, Quantitative Estimate of Drug Likeness (QED), and Ghose filter.

### 2.5 Molecular studies

#### 2.5.1 Cloning of *sufD* gene

A gene encoding *Mycobacterium tuberculosis* H37Rv *sufD (Rv1462)* was cloned into the pET-28a plasmid using NdeI and BamHI restriction sites. The *sufD* gene was amplified from *Mtb* genomic DNA in a 100 μL reaction mix (50 ng of DNA template, 0.5 mM of each primer, 0.2 mM of each dNTP, and 5 U of Taq Polymerase). The PCR conditions consisted of an initial denaturation at 95°C for 2 min, followed by 30 cycles of denaturation at 94°C for 30 s, annealing at 54°C for 30 s, and extension at 72°C for 1 min, terminating after a final extension at 72°C for 10 min. Amplified fragments were purified using a PCR cleanup kit and incubated with BamHI and NdeI enzymes at 37°C for 10 min. The digested PCR amplicons were mixed with the digested pET-28a plasmid at a 3:1 ratio in 20 μL ligation mix and incubated at 4°C overnight. 2.0 μl of ligation mix was transferred into competent *E. coli* BL21(DE3) cells by heat shock, followed by plating on LB-kanamycin selection media and overnight incubation at 37°C. We confirmed the recombinant colonies by colony PCR and restriction digestion.

Our results revealed the recombinant protein expression in inclusion bodies and was confirmed by western blot. We addressed this challenge by subcloning the *sufD* into SUMO-pRSF-Duet1 expression vector for soluble expression as this vector consists of a solubility tag SUMO in addition to the affinity tag (hexahistidine tag). Eurofins Genomics (India) sequenced the SUMO-sufD construct to check for any existing errors. This variant of recombinant SufD consists of a fused N-terminal 6x His tag followed by a SUMO peptide immediately upstream of SufD. It also includes two cleavage sites: a thrombin cleavage site between the 6x His tag and the SUMO peptide; and a SUMO protease site between the SUMO peptide and SufD polypeptide.

#### 2.5.2 Optimization of conditions for overexpression

*Escherichia coli* BL21(DE3) cells were transformed with pET-28a-sufD plasmid DNA and grown overnight at 37°C on LB-kanamycin (50 mg/ml) selection media plates. Positively confirmed recombinant colonies were assessed for pilot-scale overexpression of SufD. We conducted a series of experiments to optimize the expression conditions for the SufD, which involved varying the inducer concentrations ranging from 0.05 to 1.0 mM and employing different temperatures with different expression periods (18°C for 16 h, 25°C for 12 h, 30°C for 5 h, and 37°C for 3 h) in 10.0 mL cultures. After each experimental condition, cells were harvested, saline-washed, and the pellet was resuspended in 10.0 mL of lysis buffer (50 mM Tris–HCl pH-8, 500 mM NaCl, 10 mM β-mercaptoethanol, and 1% v/v Triton X- 100). Lysozyme was added to a final concentration of 100 μg/mL in the total cell lysate followed by an incubation period of 30 min at room temperature. The cells were ruptured by an ultrasonic probe bursting at 60% amplitude for 15 s with 15-s intervals for 30 cycles at 4°C. A subsequent centrifugation at 10,000 rpm for 30 min at 4°C separated the pellet from the supernatant of the resulting lysate and the expression profile was evaluated through SDS-PAGE for each sample.

#### 2.5.3 Purification of SufD

For overexpression of SufD, 10 ml starter culture was prepared, inoculated into 1 L LB-broth (50 mg/ml kanamycin), incubated till log-phase (OD_600_–0.6), and induced with 0.5 mM IPTG. Cells were harvested and resuspended in 60 mL of modified lysis buffer (50 mM Tris–HCl pH-8, 500 mM NaCl, 10 mM β-mercaptoethanol, 10 mM imidazole, 2% v/v Triton X-100, 2% v/v Tween-80). Lysozyme was added and cells were sonicated for 120 cycles as mentioned previously. The supernatant containing SUMO-SufD protein was separated by centrifugation, filter-sterilized, and captured on a 2 mL Ni-NTA Sepharose solid bed in a gravity flow column. The column was washed with 250 mL wash buffer A (25 mM Tris–HCl pH-8, 500 mM NaCl, 10 mM β-mercaptoethanol, 20 mM imidazole) and 150 mL of wash buffer B (25 mM Tris–HCl pH-8, 300 mM NaCl, 10 mM β-mercaptoethanol, 40 mM imidazole) to remove nonspecific proteins. The target protein was eluted out in 1 mL fractions of elution buffer (25 mM Tris–HCl pH-8.0, 300 mM NaCl, 10 mM β-mercaptoethanol, 300 mM imidazole, 10% v/v glycerol). The quantity of protein in the eluted fractions was determined by measuring absorbance at 280 nm and the purity of SUMO-SufD was checked on a 12% SDS-PAGE gel.

Affinity-purified fractions were pooled and injected into the FPLC system after equilibration of size exclusion column with 2 column volume (CV) wash of exchange buffer (20 mM Tris–HCl pH-8, 100 mM NaCl). The fractions were collected at a flow rate of 0.5 mL per minute followed by 2 CVs of wash with the exchange buffer.

Quality SEC peak fractions that appeared on SDS-PAGE were pooled and mixed with SUMO-specific protease (His-tagged Ulp1) at ratios of 25:1, 50:1, and 100:1 (substrate: protease) into 100 μl proteolytic reaction mixture. Samples were incubated at 4°C for 12 h for digestion of the N-terminal 6xHis-SUMO region, followed by SDS-PAGE analysis to determine the extent of cleavage. Properly digested samples were subjected to a second affinity purification using Ni-NTA Sepharose. 6xHis-SUMO and His-Ulp1 were captured by binding, while untagged SufD washed out into the exchange buffer. The cleaved tag and Ulp1 bound to the resin were eluted into buffer C (20 mM Tris–HCl pH-8, 100 mM NaCl, 300 mM imidazole). The approximate molecular mass and purity of the untagged SufD was determined by SDS-PAGE.

#### 2.5.4 Prediction of crystallizability

XtalPred, a web server specialized in forecasting protein crystallizability, was employed to predict the crystallization likelihood of the SufD protein. This prediction process involves comparing various protein features, amalgamating the outcomes to derive an overall probability of crystallization. XtalPred offers: (1) a thorough comparison of the protein’s characteristics against TargetDB distributions; (2) a concise summary of protein features and potential issues that might arise during crystallization attempts; (3) forecasts regarding potential ligands; and (4) optionally, listings of closely related homologs from complete microbial genomes, aiding in identifying proteins more inclined toward successful crystallization. The SufD sequence was submitted in fasta format to predict crystallizability.

## 3 Results

### 3.1 Catalog of essential, hypothetical, and non-homologous proteins

A computational subtractive genomics analysis was employed to identify essential, hypothetical, and non-homologous proteins. In the DEG database, 614 genes were identified as essential in the *Mycobacterium tuberculosis* H37Rv organism. Among these, 177 were designated as hypothetical proteins. BLASTp of *Mtb* HPs against *Homo sapiens* resulted in 152 proteins with “NoHits” (indicating non-homologous sequences), while 25 proteins produced “Hits” (indicating homologous sequences shared between the host and pathogen). For subsequent analysis, the non-homologous sequences, which exhibited no similarity with the human host, were specifically chosen. This observation prompted us to compile and create a dataset comprising essential, hypothetical proteins of *Mtb* that lack homology to human proteins, as illustrated in Supplementary Table S1.

### 3.2 Pathway prioritization

**Figure 2:**
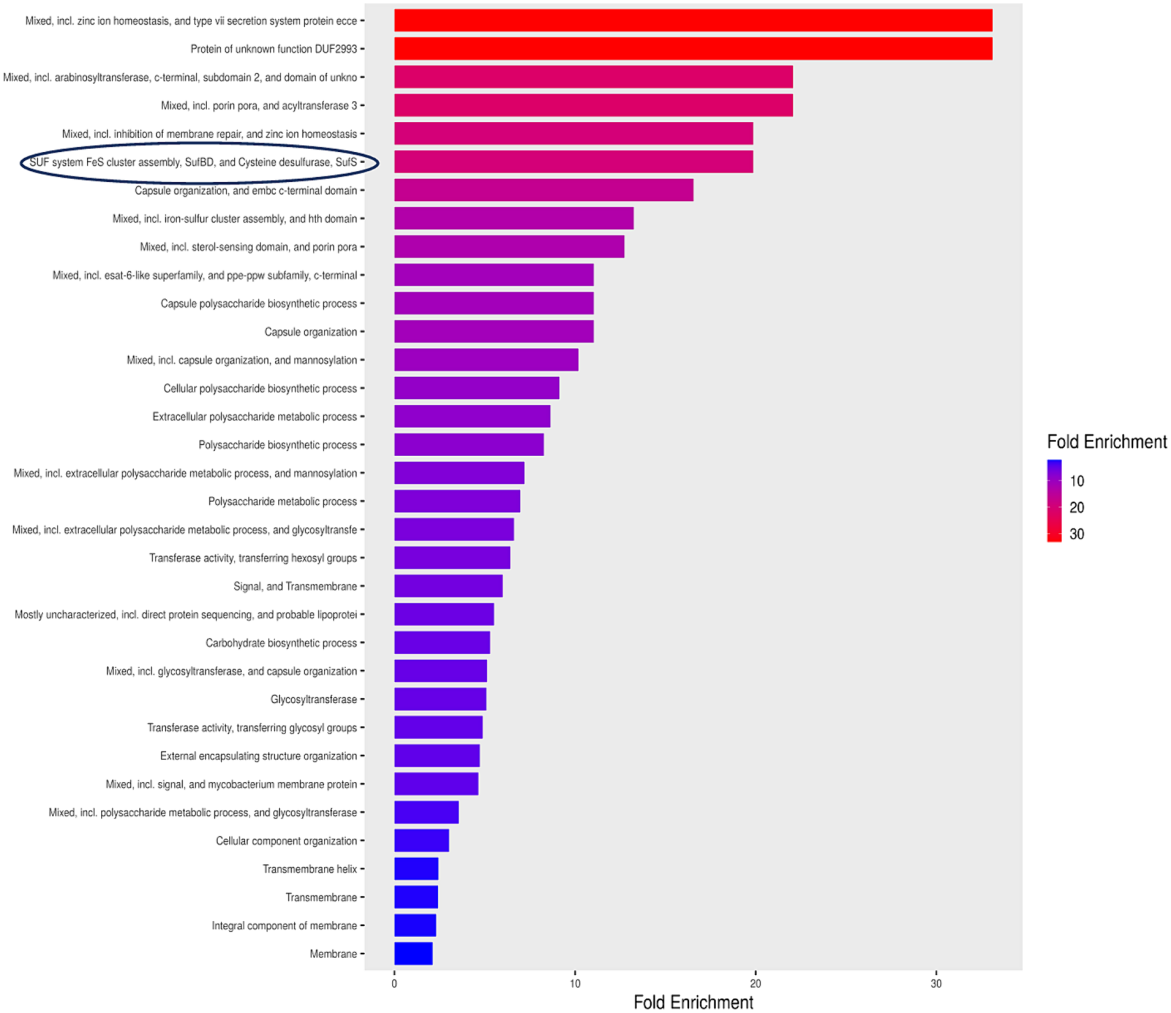
Enrichment pathway analysis for essential hypothetical proteins unique to *Mycobacterium tuberculosis*. SUF system FeS cluster assembly exhibited an enrichment surpassing 20 fold highlighted by a blue encirclement.

The investigation of 152 non-homologous proteins using Shiny GO revealed their involvement in 34 pathways. Since these pathways contain vital genes, they are being suggested as indispensable pathways for *Mtb*, as outlined in the Figure 2. The tool generated enrichment scores, where positive values signify enrichment, while negative values denote depletion. Notably, mixed pathways exhibited the top Gene Ontology terms, enriched up to 30-fold. Organizing enriched genes into functional clusters enhances our comprehension of the biological processes involved. In these pathways, particular emphasis was placed on the SUF system, responsible for FeS cluster assembly. This system demonstrated a significant 20-fold enrichment and was selected as a target pathway, revealing clusters of functionally related genes.

### 3.3 Identification of novel drug targets and significance of selected target

**Figure 3:**
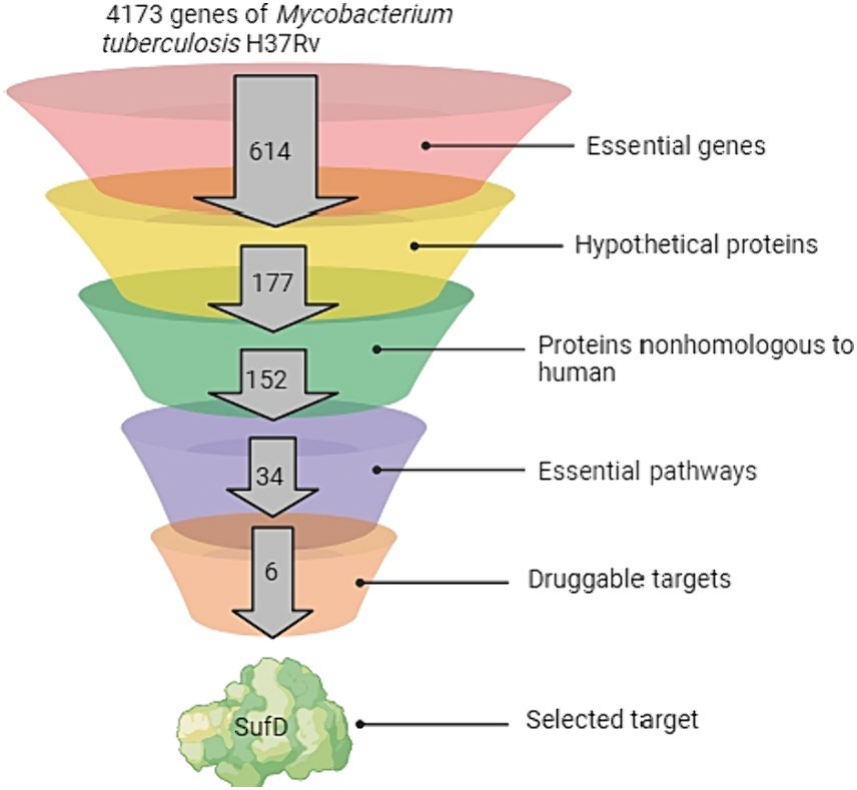
Pipeline for Target Identification. The funnel depicts the sequential analysis of the complete *Mycobacterium tuberculosis* genome at various levels.

Figure 3 showed the comprehensive outcome of the current study. Considering their essentiality, non-host nature, and involvement in vital pathways, all proteins in the SUF pathway hold promise as potential candidates for novel drug targets. Targeting any of these proteins could disrupt a pathway crucial for the pathogen’s growth and survival. In this study, SufD was specifically chosen for further investigations, while the remaining five proteins warrant attention in a separate report. SufD was annotated as a high-confidence drug target in the UniProt database. Inhibiting SufD with specific inhibitors could impede Fe–S cluster assembly, potentially inducing a bactericidal effect through the occurrence of the Fenton reaction. The significance of SufD and its central role in addressing stress conditions faced by *Mtb* underscores it as an attractive target for the design of new anti-TB drugs.

### 3.4 Conservancy and motif analysis with other species

**Figure 4:**
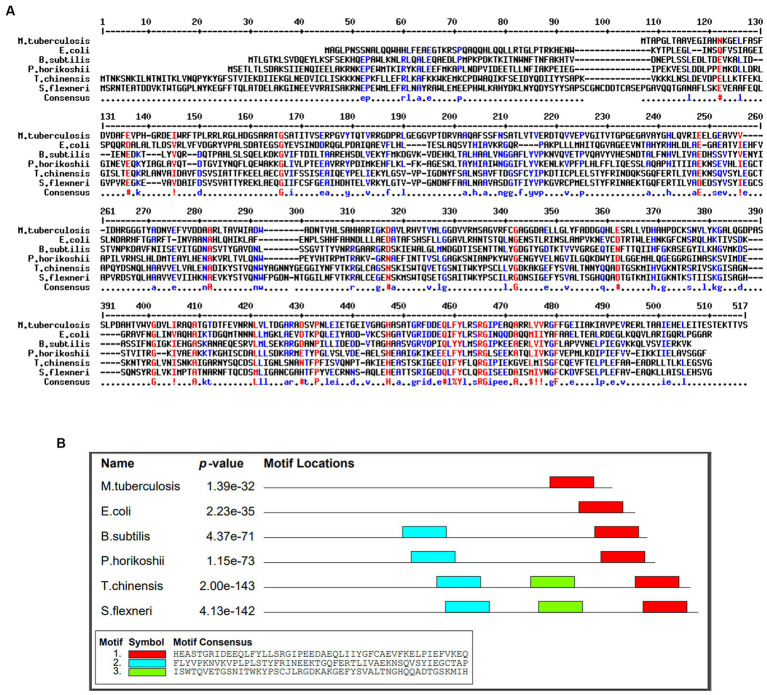
Identification of conserved residues and motifs. **(A)** Multiple sequence alignment of SufD protein sequences with selected organisms. Conserved residues are represented in red and blue. **(B)** Consenses motif identified in *M. tuberculosis* and highlighted in red color.

A conservancy analysis was conducted on the SufD protein sequence. The BLASTp search for SufD produced local alignments with homologous proteins, including P77689 (*Escherichia coli K-12*), O32165.1 (*Bacillus subtilis*), O50093.1 (*Pyrococcus horikoshii*), P49530.1 (*Trieres chinensis*), and Q83KW2.1 (*Shigella flexneri*). These hits underwent additional analysis through multiple sequence alignment using MULTALIN, where highly conserved residues are represented in red, and weakly conserved residues in blue as illustrated in Figure 4A. The alignment revealed conserved domains and motifs among these proteins. Protein motifs are indicative of active sites, help to identify regions influencing protein structure and stability, and aid in classifying proteins into families. Uncovering sequence motifs contributes to a more profound understanding of molecular structure and function. The homologous sequences were further scrutinized for motifs, leading to the identification of the consensus motif sequence HASATGRFDDEQLFYLRSRGIPEAQARRLVVRGFFGEIIAKIAVPE VRER in *Mtb* as depicted in Figure 4B. Additionally, a correlation was observed between the conserved region identified in the multiple alignment and the consensus motif, reinforcing the significance of these conserved elements.

### 3.5 Subcellular location prediction

Understanding the sub-cellular localization of a drug target is crucial for optimizing the drug’s mode of action against specific target. Numerous examples in the literature highlight cytoplasmic proteins as effective therapeutic targets due to their accessibility to drugs. The prediction of the sub-cellular localization of SufD was accomplished using computational tools, specifically PSORTb and CELLO. The results indicate that the protein is localized in the cytoplasm.

### 3.6 Structure prediction and validation

**Figure 5:**
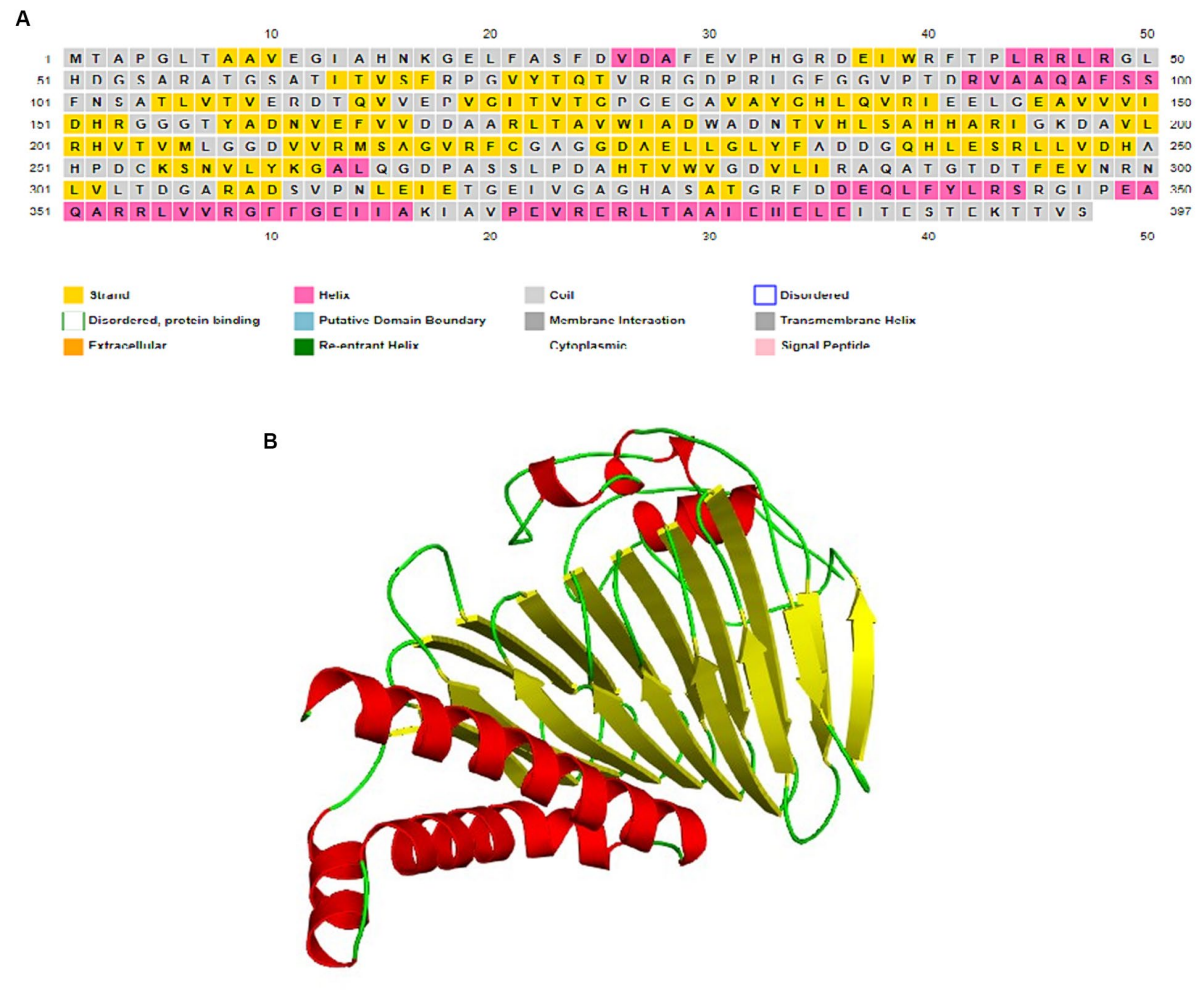
Structure prediction. **(A)** Secondary structure prediction by PSIPRED server. **(B)** Predicted 3D structure of SufD rendered by PyMOL.

I-TASSER server utilized the TM-align structural alignment program to identify optimal templates in the PDB library. Tertiary structure of SufD was predicted by I-TASSER and reported five models. The confidence of each model was quantitatively assessed using the C-score. The most suitable model for SufD exhibited a C-score of −3.51, falling within the acceptable range of [−5, 2] and was depicted in Figure 5B. Various tools were employed to validate the modeled protein structure. PROCHECK generated a Ramachandran plot for the modeled protein structures, revealing approximately 93.8% of residues in the favorable region, 6.2% in additionally allowed regions, and 0% in generously allowed regions, with no residues in disallowed regions, as depicted in the Supplementary Figure S1. PSIPRED predicted the formation of alpha helices and beta sheets, as illustrated in the Figure 5A.

### 3.7 Functional characterization

The ProFunc tool was used to validate the functional aspects of the protein through structural analysis. The predicted protein structure revealed the presence of a functional domain in the Pfam database, identified by the Pfam domain ID PF01458. This particular domain holds significance in the SUF system, playing a vital role in the assembly of Fe–S clusters.

### 3.8 Prediction of residues in binding pockets

To facilitate the exploration of ligand binding within the modeled protein structure, the CastP tool was utilized to identify interactive binding cavities. According to CASTp’s characterization of binding sites, the foremost predicted pocket exhibited a surface area of 686.2 Å^2^ and a spatial volume of 553.2 Å^3^ while other pockets had surface areas less than or equal to 100 Å^2^. Initial CAST analysis of over 100 proteins revealed that the largest pocket/cavity is often the active site. This criterion formed the basis for selecting the first pocket for further binding studies. The predicted pocket was observed to encompass residues V10, E11, G12, I13, A14, H15, K17, G18, E19, L20, F21, A22, A28, F29, E30, V31, P32, H33, D36, E37, I38, W39, R40, F41, G86, G87, V88, T90, D91, R92, A94, A189, H190, H191, A192, R213, S214, A215, G217, V218, R219, R244, L245, L246, D248, V280, D282, V312, N314. The binding site of SufD was also determined by SCFBio active site prediction server and interestingly, the predicted cavity was found to share the above amino acid residues.

### 3.9 Selection of best docked compounds

The compounds ranking within the top 12, each possessing binding scores surpassing −100 kcal/mol, have been recognized as potential binders and are outlined in Table 1. All selected ligands have established connections with binding site residues and demonstrate markedly elevated binding scores when compared to the control compound (CHEMBL1539876). The notably high negative scores indicate superior binding capabilities of these compounds, suggesting their potential to form robust and stable complexes with the target. Additionally, the chemical structures and molecular formulas of the top 12 compounds with the best docking are included in Table 2.

**Table 1:** Binding energies of ChEMBL compounds with SufD.

**ChEMBL ID**	**Moldock score**	**Rerank score**
CHEMBL1539876*	−129.76	−98.76
ChEMBL3677601	−204.88	−121.43
ChEMBL3893839	−200.38	−155.35
ChEMBL4109740	−184.78	−135.694
ChEMBL3651495	−174.45	−101.03
ChEMBL3921511	−166.02	−112.63
ChEMBL3908295	−161.68	−123.906
ChEMBL3912062	−156.15	−110.2
ChEMBL3697959	−157.91	−128.18
ChEMBL3718414	−154.8	−101.24
ChEMBL3716905	−146.02	−99.77
ChEMBL3962891	−144.89	−116.17
ChEMBL3915781	−138.61	−118.73

*Control.

### 3.10 Evaluation of ligands

Table 2 presents the drug-likeness properties of the top-docked compounds. While CHEMBL3677601 and CHEMBL3893839 were found to violate Lipinski’s rule, CHEMBL4109740 adhered to the rule with a logP value of ≤5, a number of hydrogen bond donors ≤5, and a number of hydrogen bond acceptors ≤10. The AlogP value, indicating the compound’s hydrophilicity, was less than 5. Consequently, CHEMBL4109740 was chosen for ADMET analysis, and the results obtained from the admetSAR server are displayed in Table 3. An ideal oral drug should be absorbed effectively from the gastrointestinal tract, specifically target the desired site, undergo metabolism without losing its properties, and be eliminated without causing harm. CHEMBL4109740 demonstrated good intestinal absorption, non-substrate and non-inhibitor status for p-glycoprotein, non-AMES toxicity, and non-carcinogenic properties.

**Table 2:** Drug likeliness prediction.

**CHEMBLID**	**MW**	**logp**	**Alogp**	**HBA**	**HBD**	**TPSA**
CHEMBL15398768*	A	3.08	0.391	4	2	53.85
CHEMBL3677601	643.2	1.43	2.43	10	2	204.12
CHEMBL3893839	558.2	−0.26	−3.57	12	3	158.65
CHEMBL4109740	515.1	0.92	−0.21	8	3	145.14
CHEMBL3651495	450.1	2.18	1.078	6	0	94.27
CHEMBL3921511	484.2	0.88	1.863	7	1	93.51
CHEMBL3908295	411.2	−2.59	−1.98	9	1	109.82

*Control.

**Table 3:** ADMET Predicted Profile for CHEMBL4109740.

**Model**	**Result**	**Probability**
**Absorption**		
Blood–brain barrier	BBB+	0.506
Human intestinal absorption	HIA+	0.896
Caco-2 permeability	Caco2-	0.6512
P-glycoprotein substrate	Non-substrate	0.6041
P-glycoprotein inhibitor	Non-inhibitor	0.6714
Renal organic cation transporter	Non-inhibitor	0.8401
**Distribution**		
Subcellular localization	Mitochondria	0.4066
**Metabolism**		
CYP450 2C9 substrate	Non-substrate	0.7858
CYP450 2D6 substrate	Non-substrate	0.7891
CYP450 3A4 substrate	Non-substrate	0.5051
CYP450 1A2 inhibitor	Non-inhibitor	0.5439
CYP450 2C9 inhibitor	Inhibitor	0.6052
CYP450 2D6 inhibitor	Non-inhibitor	0.8151
CYP450 2C19 inhibitor	Inhibitor	0.6176
CYP450 3A4 inhibitor	InhibitorHigh CYP Inhibitory	0.8497
CYP inhibitory promiscuity	Promiscuity	0.9373
**Toxicity**		
Human ether-a-go-go-related gene inhibition	Weak inhibitor	0.8302
AMES toxicity	Non AMES toxic	0.5955
Carcinogens	Non-carcinogens	0.6483
Fish toxicity	High FHMT	0.9869
Tetrahymena pyriformis toxicity	High TPT	0.9422
Honey bee toxicity	Low HBT	0.7814
Biodegradation	Not ready biodegradable	0.9933
Acute oral toxicity	III	0.5768
Carcinogenicity (Three-class)	Non-required	0.576

### 3.11 Identification of residues involved in interaction

Analyzing the interactions with crucial amino acid residues is essential to validate if the ligand is docked in a favorable conformation. These interactions are pivotal in energetically stabilizing the ligand at the macromolecule structure’s juncture. The proximity of a lead molecule to the binding site of the target frequently suggests increased biological efficacy compared to a molecule situated farther away.

**Figure 6:**
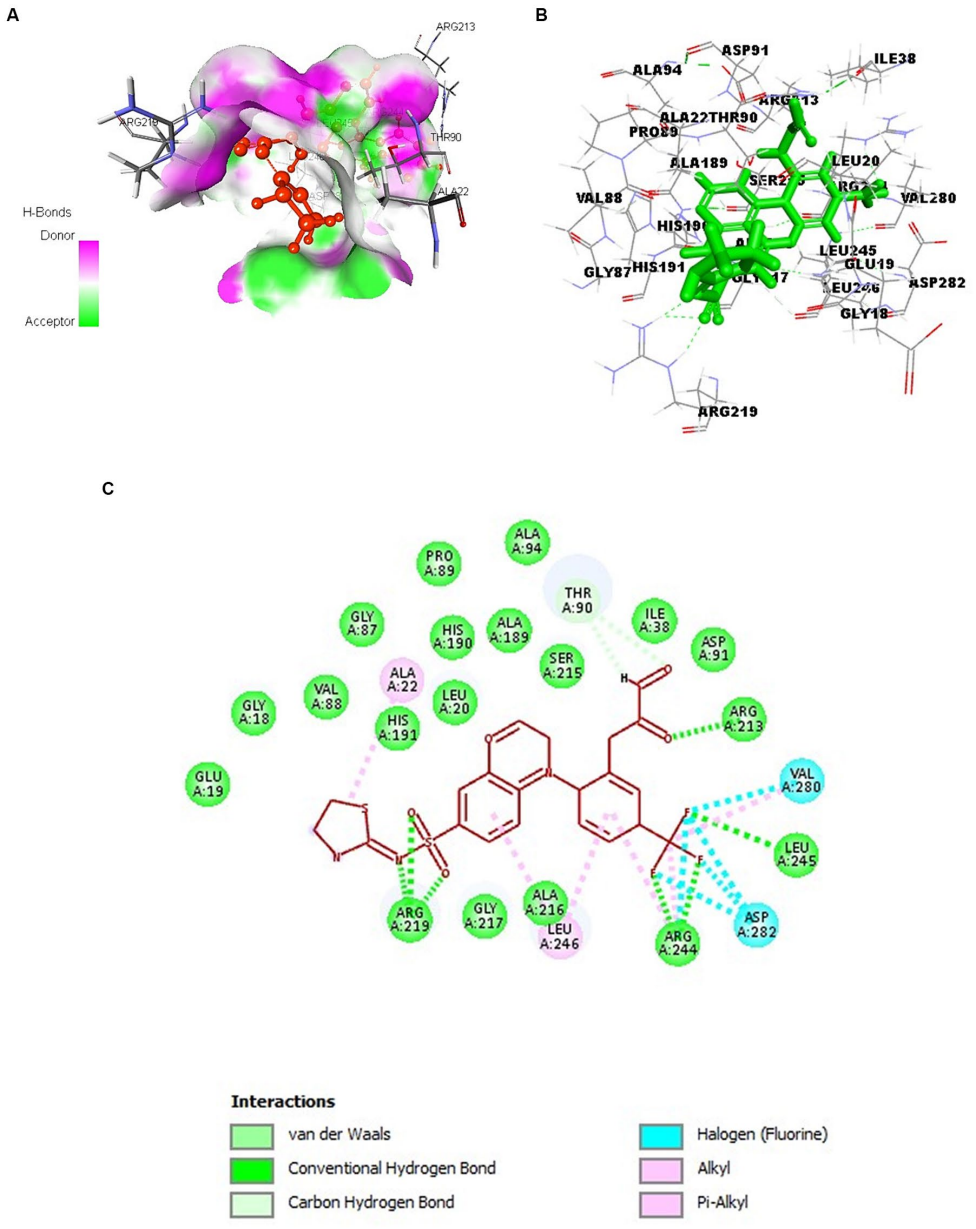
Docking of CHEMBL4109740 compound with SufD. **(A)** Hydrogen bond interactions of ligand (red) at the binding pocket of protein. **(B)** 3D structure view of molecular docking of ligand (green sticks) with protein residues. **(C)** 2D interactions of ligand (red) with aminoacid residues of protein.

Figures 6A–C illustrates various amino acid interactions, including hydrogen, halogen, electrostatic, and alkyl interactions between SufD and the optimal compound, CHEMBL4109740. Specifically, the compound engages in hydrogen bond interactions with amino acid residues Arg 213, Arg 219, Arg 244, and Leu 245. Additionally, Val 280 and Asp 282 participate in halogen interactions, Thr 90 in carbon-hydrogen bond interactions, and Ala 22 and Leu 246 in alkyl interactions.

### 3.12 Cloning of *sufD* gene in expression vector

**Figure 7:**
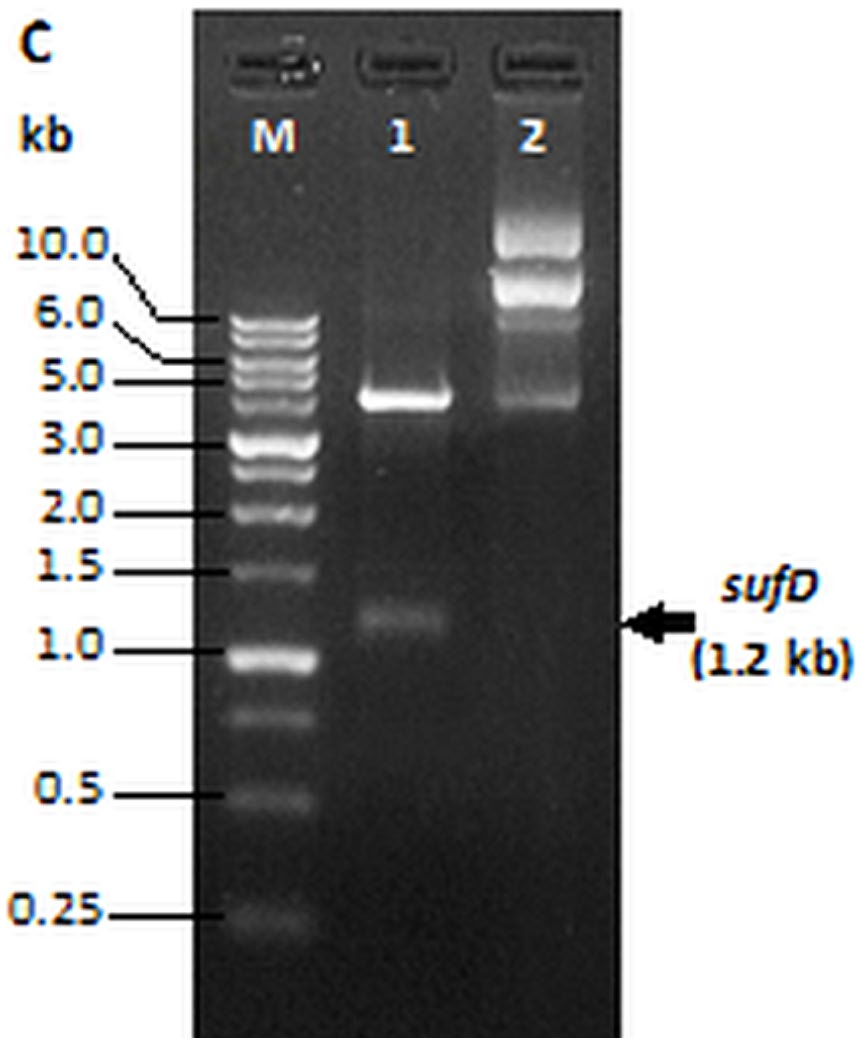
Restriction digestion of SUMO-*sufD* vector. Lane 1- SUMO-sufD digested with BamHI and XhoI, lane 2- undigested SUMO plasmid. Lane M-1 kb DNA ladder (PUREGENE™ PG010-500DI-NV).

Amplification of *sufD* from pET28a-sufD plasmid with the gene-specific primer pair designed for sub-cloning produced a 1.2 kb band. The PCR product that is digested and ligated in SUMO-pRSF-Duet1 vector resulted into SUMO-sufD construct of 5.1 kb. BamHI and XhoI digestion of this construct released 1.2 kb insert corresponding to the target gene as observed in Figure 7 confirming the successful subcloning of the *sufD* in SUMO expression vector.

### 3.13 Optimal conditions for expression of SufD

We systematically screened conditions, such as inducer concentration, post-induction incubation temperature, and duration of enhanced protein expression and solubility. In the inducer concentration optimization, we observed the highest expression with a concentration of 0.5 mM IPTG. Subsequent optimization of post-induction temperature and expression duration revealed high protein expression at a temperature of 18°C and incubation time of 16 h. Despite our optimization efforts, pET28a-SufD was consistently found in inclusion bodies in all the experiments and confirmed by western blot as depicted in Supplementary Figure 2. This indicates its challenging nature for achieving soluble expression when heterologously expressed *in E. coli*.

Combining all the optimal conditions, we expressed SUMO-SufD by inducing it at 18°C with 0.5 mM IPTG for 16 h. Expression was checked by loading an equal amount of uninduced and induced supernatants of crude extract on 12% SDS-PAGE. Under these conditions a significant proportion of SufD was successfully expressed in the soluble fraction which is indicated by a strong band of target protein.

### 3.14 Purification of SufD by affinity and size exclusion chromatography

**Figure 8:**
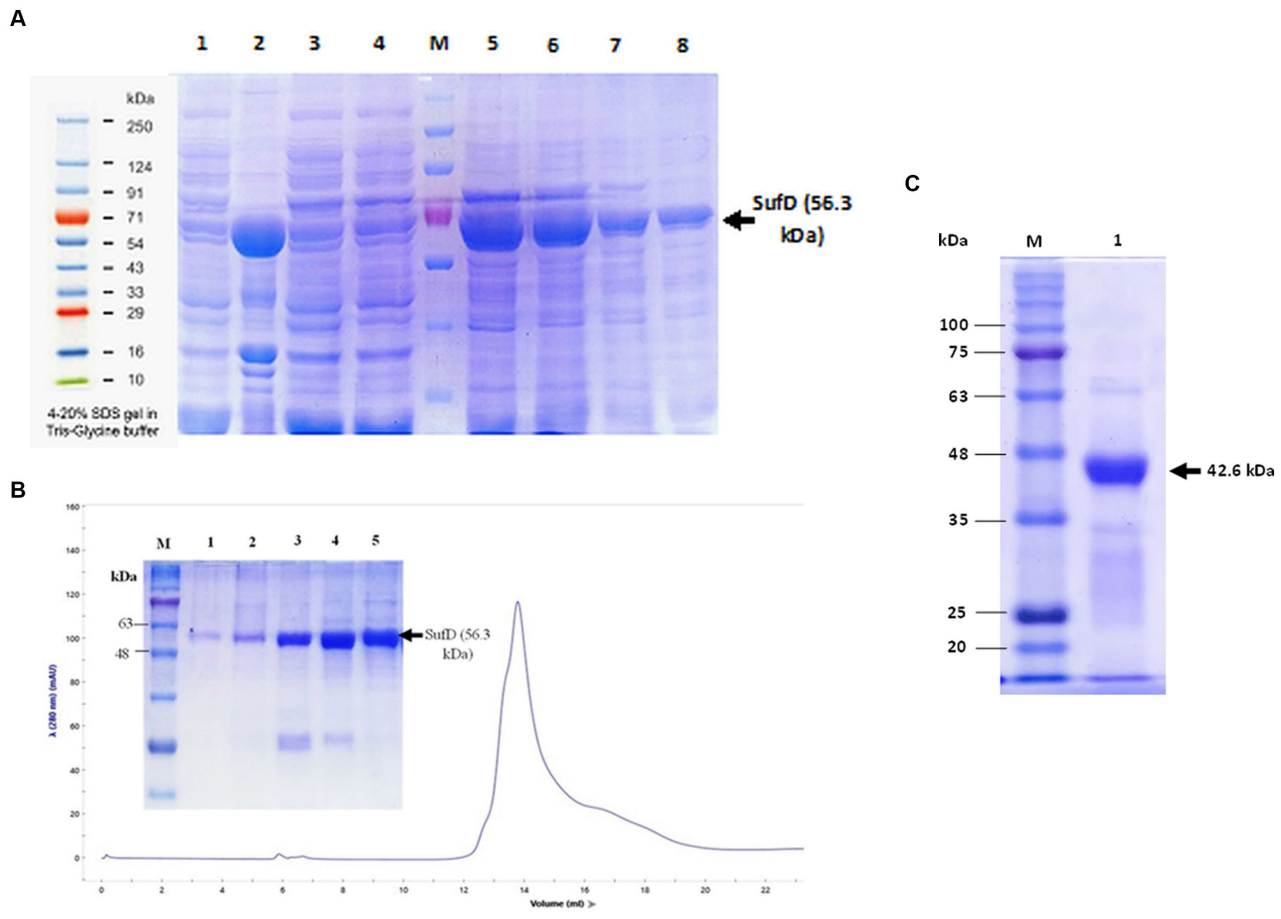
Purification of SufD. **(A)** Affinity chromatography. Lane 1- uninduced supernatant, lane 2-induced supernatant, lane 3- flow-through, lane 4- wash fraction, lane 5, 6, 7, 8- elutions. **(B)** Size exclusion chromatography: SufD protein shows the UV_280 nm_ absorbance profile (blue line) and SDS-PAGE gel of different fractions eluted from the column and distributed along the chromatographic peak (lane 1–5). **(C)**. Second IMAC purification: Lane 1- pure untagged SufD Lane M-molecular mass marker.

SufD was purified using Nickel-NTA column affinity chromatography and analysis of all the fractions through SDS-PAGE confirmed the presence of SufD at the expected size of 56.3 kDa as shown in Figure 8A. Consequently, the hexahistidine SUMO fusion SufD construct demonstrated efficient solubilization. To further enhance protein quality, eliminate salts and aggregates, size exclusion chromatography was conducted before proceeding with the cleavage of fusion tags. A single SEC peak in the chromatogram suggested that the eluted protein was monomeric and homogenous as shown in Figure 8B. SDS-PAGE gel from peak fractions displayed 56.3 kDa bands confirming that SufD proteins are intact with the highest purity. Ulp1 proteolysis cleaved the SUMO tag after G120-G121 residues, leaving only one residue (S122) before the original SufD polypeptide (M123-S519). Notably, a substrate-to-protease ratio of 100:1 (w/w) was sufficient for complete digestion when incubated at 4°C for 12 h. The second affinity purification allowed convenient isolation of the target protein in its pure and native form, as demonstrated in Figure 8C.

### 3.15 Prediction of crystallizability parameters

**Figure 9:**
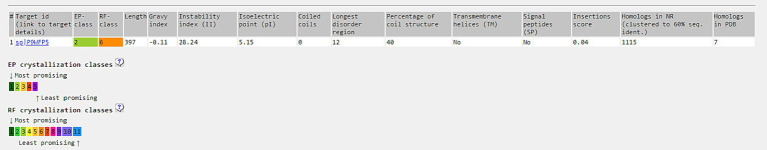
Prediction of crystallizability with Xtalpred server.

XtalPred categorizes proteins into five “crystallization classes” through a statistical examination of the physicochemical attributes of a protein. Parameters such as an EP value of 2 and an RF value of 6 for SufD are indicative of favorable conditions for crystallization. Additionally, it was predicted to have an isoelectric point of 5.5, a disorder region of 12 residues, and a stability index of 28.24 suggests protein was stable. Moreover, the absence of transmembrane helices, and signal peptides is a positive indicator for the potential success of crystallization. All these parameters were illustrated in Figure 9.

## 4 Discussion

The recrudescence of tuberculosis and the emergence of antibiotic resistance have compelled the identification of potential targets and rapid development of new antituberculosis drugs across the globe. Complete eradication of dormant *Mtb* necessitates inhibiting stress response pathways (Krug et al., 2023). Several hypothetical proteins are predicted to be pivotal for *Mtb*’s intracellular lifestyle, aiding its survival in diverse environments. Roughly a quarter of *Mtb* genes are annotated as hypothetical proteins (Yang H. et al., 2019; Yang Z. et al., 2019). Ideally, a drug target should be essential to the pathogen’s growth and non-homologous to the host to avoid cross-reactivity and adverse effects. These proteins hold significant importance due to their involvement in various cellular processes, which could offer critical insights into understanding the disease mechanisms (Hasan et al., 2006). Among the 4,173 genes of *Mtb*, 614 genes were designated as essential genes in the DEG database, and these genes have been identified as vital through the transposon site hybridization approach (Sassetti et al., 2003).

In the quest to pinpoint distinctive and promising druggable targets within *Mtb*, we employed the subtractive genomics method to prioritize potential drug targets. Hundred and fifty-two proteins were identified as non-homologous to human among the 177 essential genes (categorized as hypothetical proteins). Focusing on the pathogen’s biosynthetic pathways to locate potential drug targets proves advantageous, as each stage within these pathways is critical for the pathogen’s survival and proliferation. Consequently, delving deeper into the metabolic pathway analysis of these proteins led to the prediction of 34 essential pathways. This insight highlighted the genes contributing to enrichment, facilitating a more thorough analysis, observing a 20-fold enrichment in the SUF pathway. As a result, the Suf proteins, responsible for Fe–S cluster assembly, emerged as promising drug candidates (Vernis et al., 2017). Notably, previous research has established SufD’s crucial role in iron acquisition in *E. coli* (Saini et al., 2010). The strain with a mutated *sufD* gene in *S. aureus* displayed various phenotypic traits linked to compromised maturation of Fe–S proteins. These traits encompassed reduced activities of enzymes dependent on Fe–S clusters, lowered flux through the tricarboxylic acid (TCA) cycle, susceptibility to reactive oxygen and nitrogen species, elevated DNA damage, and impaired DNA repair mechanisms. Additionally, this strain demonstrated disruptions in intracellular nonchelated iron pools (Roberts et al., 2017). Expanding upon these findings, we propose a hypothesis that in *Mtb*, SufD plays a key role in capturing ferrous ions released during Fe–S cluster damage, thereby preventing the Fenton reaction. The SufD was specifically chosen for further characterization and exploration as a potential drug target against *Mtb*, while the remaining five proposed drug targets remain viable for future studies.

The other important objective of this study was the *in silico* characterization of SufD. Annotation of proteins aids in understanding and cataloguing various aspects of a protein’s structure, function, and other relevant characteristics. Annotations typically include functional, structural, and domain annotation. In molecular biology and bioinformatics research fields, the utilization of multiple sequence alignments (MSAs) has become a foundational method. This includes the identification of conserved regions for three-dimensional (3D) structure prediction, and the clarification of molecular function. Various bioinformatics tools enabled the identification of conserved regions and motifs. It is widely accepted that significant sequence similarity is generally a good indicator of similarity in function (Joshi and Xu, 2007). Tracing the location of protein not only aids in understanding its function but also influences the design of novel drugs and vaccines in drug discovery. Cell membrane proteins are often targeted for vaccines, whereas cytoplasmic proteins are commonly targeted for therapeutic purposes (Anis Ahamed et al., 2021). Localization of SufD in cytoplasm makes it an attractive therapeutic target.

The SufD protein structures were searched using BLASTp against the PDB database. As the 3D structure was unavailable in the PDB, we used the I-TASSER tool for its homology modelling. Assessment of modeled structure via the Ramachandran plot revealed that most residues were present in the acceptable or favored areas, with a few residues in additional allowed regions and none in the disallowed regions. The structural characteristics of proteins determine a myriad of functions, ranging from conferring binding specificity and mechanical stability to catalyzing biochemical reactions, facilitating transport, and transmitting signals. Therefore, structure-based function prediction significantly enhances functional annotation (Stollar and Smith, 2020). In the case of SufD, we observed it to possess a PF01458 functional domain, affirming our sequence-based analysis. With the structural modelling completed, the focus shifted to locating the active site where a ligand could potentially bind, altering its function. The CASTp server provided insight into the amino acid residues present within the protein’s binding pocket.

Molecular docking was employed for inhibitor screening, evaluating 147 docked compounds. Among them, 12 displayed superior dock scores compared to the control compound (ChEMBL1539876). In the prior study by Choby et al., control compound was denoted as the ‘882 molecule. The study elucidated that ‘882 disrupts the assembly of Fe–S clusters in apo-proteins, a pivotal process in *S. aureus*. A comprehensive analysis involving genetic, physiological, and biochemical investigations indicates that ‘882 primarily inhibits iron–sulfur (Fe–S) cluster assembly, by targeting the Suf complex. These experiments in *S. aureus* support our *in silico* findings for SufD, as the shortlisted compounds demonstrated higher binding affinity than the 882 molecule. Subsequent interaction analysis was focused on the best compounds using Biovia Discovery Studio. Considering the Lipinski Rule of Five and the molecular profile, the top 6 compounds were scrutinized for drug probability, leading to the selection of the most promising candidate for ADMET profiling. Assessing the ADMET properties is crucial in gauging a drug candidate’s behavior, potential toxicity, and fate within the human body (Ferreira and Andricopulo, 2019). When subjected to ADMET profiling, the chosen compound demonstrated favorable characteristics, displaying no adverse effects on absorption and exhibiting no cytotoxic, hepatotoxic, or mutagenic properties.

SufD was predicted to be a potential drug target through an *in silico* target identification approach, making it an important protein to study. Given this, we cloned the sufD, expressed, and purified rSufD in *E. coli*. Even though heterologous protein expression in the *E. coli* system is well-established, the optimal scheme varies for each target protein. The objective for optimization was to attain the highest soluble production of SufD in the shake flask culture. Hence, we investigated the effect of inducer concentrations on the expression of SufD and found that 0.5 mM was the optimum IPTG concentration. We also examined the influence of different post-induction temperatures and incubation times on SufD expression. According to our results, a reduction of the temperature down to 18°C along with post-induction incubation for 16 h enhanced the protein expression, but it formed inclusion bodies and was confirmed by western blot. We addressed this challenge by subcloning the *sufD* into SUMO-pRSF-Duet1 expression vector for soluble expression as this vector consists of a solubility tag SUMO, in addition to the affinity tag (hexahistidine tag) (Butt et al., 2005). Incorporating all the optimal conditions together, SufD was expressed in the soluble fraction when induced with 0.5 mM IPTG and was incubated at 18°C for 16 h.

Subsequent purification of rSufD by Ni-NTA affinity chromatography and SDS-PAGE analysis of all corresponding fractions revealed the SufD at an expected size of 56.3 kDa. Protein was immediately subjected to size exclusion chromatography to remove salts and aggregates before fusion tag removal. Proteolytic cleavage of the tag is usually necessary as it interferes with the structural or functional properties of the recombinant protein. Ulp1 digested the protein successfully and verified by SDS-PAGE, revealing a 42.6 kDa band corresponding to the target protein and a 13.7 kDa band corresponding to the 6xHis-SUMO tag. The SufD was subjected to a second purification step by affinity column chromatography utilizing Ni-NTA resin. Subsequently, this purification was validated through SDS-PAGE, revealing the presence of a singular, distinct band of purified de-tagged SufD. Our findings suggest that the SUMO fusion system is a promising strategy for other challenging proteins of *Mtb,* facilitating soluble expression and purification in *E. coli*.

The present study comprehensively addressed key and impactful pharmacological targets in *Mtb*, offering valuable insights for further experimental studies. Moreover, we have also identified promising inhibitor for SufD in *Mtb* which can possibly disrupt the Fe–S complex similar to 882 molecule in *S. aureus*. This combinatorial approach holds promise for the development of potential therapeutic strategies not only targeting *Mtb* but also extending to other pathogens.

## 5 Conclusion

This study addresses the desperate need for novel targets and antituberculosis drugs in response to the rise of antibiotic resistance. Our research navigated through a subtractive genomics method, highlighting 177 essential genes among hypothetical proteins, ultimately narrowing down to SufD as a potential drug target. The study also focused on the characterization of SufD, involving protein annotation from sequence-based analysis to homology modelling and structural validation confirming the presence of functional domain. Subsequent exploration of the protein’s binding pocket and inhibitor screening through molecular docking identified promising compounds with favorable drug-like properties. The best compound showed no adverse effects on absorption, cytotoxicity, hepatotoxicity, or mutagenicity. The study did not solely rely on computational methods. We ventured into the practical realm by successfully cloning, expressing, and purifying SufD in a soluble form using the SUMO fusion system, overcoming challenges associated with heterologous protein expression in *E. coli*. The findings presented here provide a roadmap for the crystallization of SufD and subsequent experimental validation of these compounds against the target. SufD inhibitors, in combination with cornerstone TB drugs and ROS-inducing antibiotics, may have synergistic effects in combating multidrug-resistant tuberculosis. This combinatorial approach signifies a potential breakthrough in targeting *Mtb* and sets a precedent for tackling other pathogens.

## Data availability statement

The datasets presented in this study can be found in online repositories. The names of the repository/repositories and accession number(s) can be found in the article/Supplementary material.

## Author contributions

NG: Conceptualization, Investigation, Methodology, Software, Visualization, Data curation, Formal analysis, Validation, Writing – original draft, Writing – review & editing. AB: Methodology, Data curation, Formal analysis, Validation, Writing – review & editing. SS: Conceptualization, Funding acquisition, Project administration, Supervision, Resources, Investigation, Methodology, Data curation, Formal analysis, Validation, Writing – original draft, Writing – review & editing.
