# Supplementary material for: Genome mining of Mycobacterium tuberculosis: targeting SufD as a novel drug candidate through in silico characterization and inhibitor screening
Source: Front Microbiol. 2024 Apr 15;15:1369645. doi: 10.3389/fmicb.2024.1369645 (PMC11057465; doi:10.3389/fmicb.2024.1369645)

SUPPLEMENTARY MATERIAL

Genome Mining of *Mycobacterium tuberculosis*: Targeting SufD as a Novel Drug Candidate through*In-silico* Characterization and Inhibitor Screening

Neelima Gorityala[a], Anthony Samit Baidya[a], Someswar R. Sagurthi[a*]

a. Department of Genetics and Biotechnology, Osmania University, Hyderabad, Telangana, India

Supplementary Table 2

Chemical structures of best-docked compounds

ChEMBL ID	2D structure	Molecular formula
ChEMBL1539876*	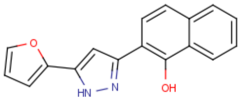	$C_{17}H_{12}N_2O_2$
ChEMBL3677601	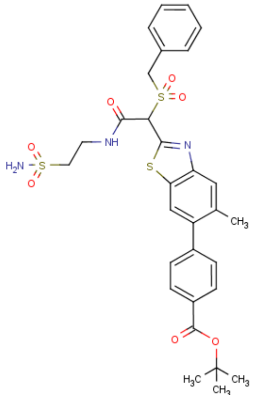	$C_{30}H_{33}N_3O_7S_3$
ChEMBL3893839	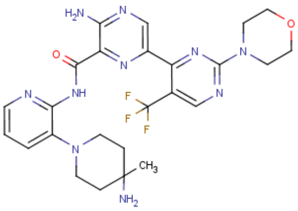	$C_{25}H_{29}F_3N_{10}O_2$
ChEMBL4109740	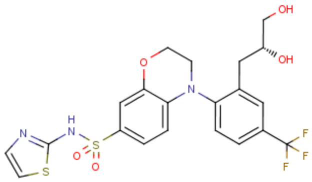	$C_{21}H_{20}F_3N_3O_5S_2$
ChEMBL3651495	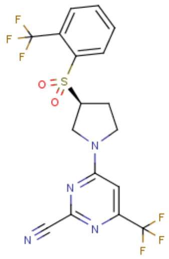	$C_{17}H_{12}F_6N_4O_2S$

ChEMBL3921511 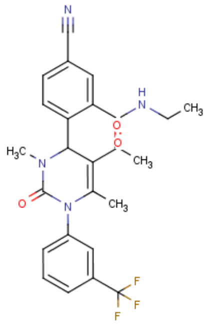 $C_{25}H_{23}F_3N_4O_3$

ChEMBL3908295 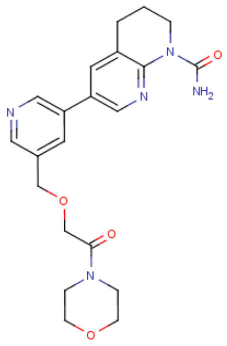 $C_{21}H_{25}N_5O_4$

ChEMBL3912062 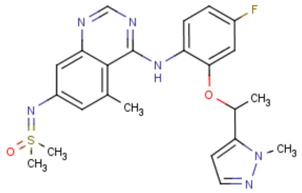 $C_{23}H_{25}FN_6O_2S$

ChEMBL3697959 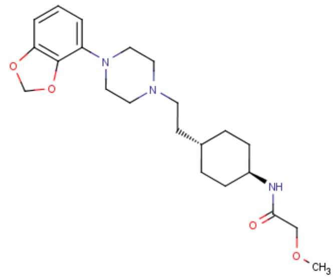 $C_{22}H_{33}N_3O_4$

ChEMBL3718414	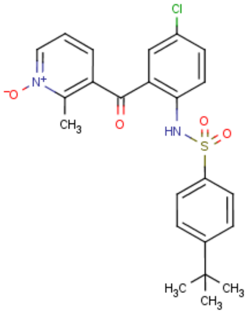	$C_{23}H_{23}ClN_2O_4S$
ChEMBL3716905	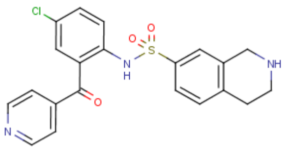	$C_{21}H_{18}ClN_3O_3S$
ChEMBL3962891	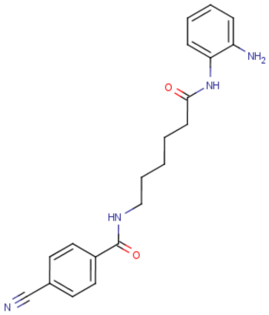	$C_{20}H_{22}N_4O_2$
ChEMBL3915781	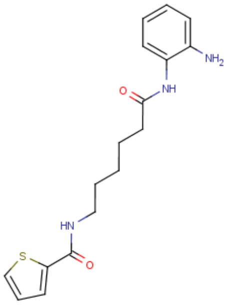	$C_{17}H_{21}N_3O_2S$

*Control :ChEMBL1539876

Supplementary Figure 1

Evaluation of predicted structure. Ramachandran plot depicting 93.8% residues in most favoured regions, 6.2% residues in additional allowed regions and 0% residues in disallowed regions

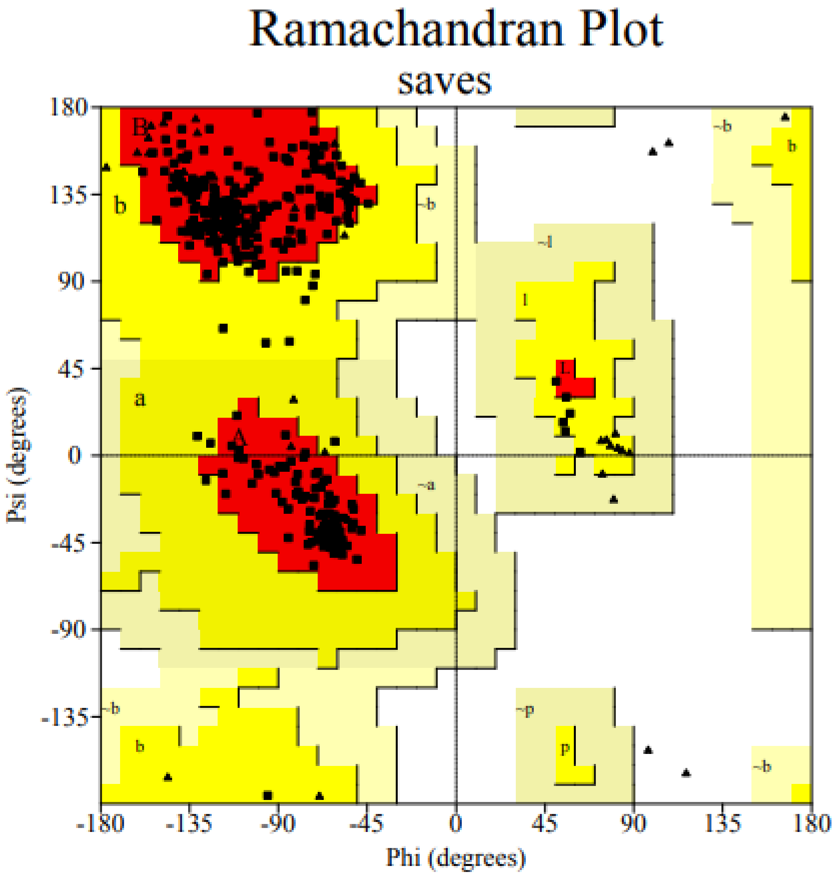

Supplementary Figure 2

Confirmation of SufD in inclusion bodies with SDS-PAGE and Western blot analysis: In both the images   lane 1- insoluble pellet, lane 2- supernatant, lane 3- solubilized pellet, lane 4- Ni-NTA purified SufD from inclusion bodies, lane 5-dialyzed protein after purification. Lane M- 10~250 kDa protein ladder (PUREGENETM PG-PMT2922).

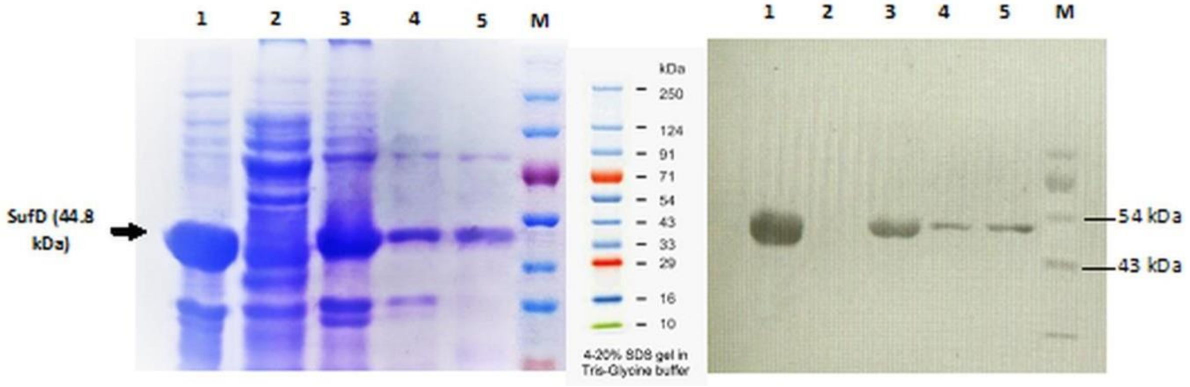

Supplement: Supplementary file 1 [file Data_Sheet_1.PDF]
